# Effects of polygenic risk for major mental disorders and cross-disorder on cortical complexity

**DOI:** 10.1017/S0033291721001082

**Published:** 2022-12

**Authors:** Simon Schmitt, Tina Meller, Frederike Stein, Katharina Brosch, Kai Ringwald, Julia-Katharina Pfarr, Clemens Bordin, Nina Peusch, Olaf Steinsträter, Dominik Grotegerd, Katharina Dohm, Susanne Meinert, Katharina Förster, Ronny Redlich, Nils Opel, Tim Hahn, Andreas Jansen, Andreas J. Forstner, Fabian Streit, Stephanie H. Witt, Marcella Rietschel, Bertram Müller-Myhsok, Markus M. Nöthen, Udo Dannlowski, Axel Krug, Tilo Kircher, Igor Nenadić

**Affiliations:** 1 Department of Psychiatry and Psychotherapy, Philipps-Universität Marburg, Rudolf-Bultmann-Str. 8, 35039 Marburg, Germany; 2 Center for Mind, Brain and Behavior (CMBB), Philipps-Universität Marburg and Justus Liebig Universität Giessen, Hans-Meerwein-Str. 6, 35032 Marburg, Germany; 3 Marburg University Hospital – UKGM, Rudolf-Bultmann-Str. 8, 35039 Marburg, Germany; 4 Department of Psychiatry, Westfälische Wilhelms-Universität Münster, Albert-Schweitzer-Campus 1, Building A9, 48149 Münster, Germany; 5 Department of Psychology, University of Halle, Halle, Germany; 6 Faculty of Medicine, Core-Facility BrainImaging, Philipps-Universität Marburg, Rudolf-Bultmann-Str. 8, 35039, Germany; 7 Centre for Human Genetics, Philipps-Universität Marburg, Baldingerstr., 35033 Marburg, Germany; 8 Institute of Human Genetics, University of Bonn, School of Medicine & University Hospital Bonn, Venusberg-Campus 1, 53127 Bonn, Germany; 9 Institute of Neuroscience and Medicine (INM-1), Research Center Jülich, Wilhelm-Johnen-Straße, 52428 Jülich, Germany; 10 Department of Genetic Epidemiology in Psychiatry, Central Institute of Mental Health, Medical Faculty Mannheim, Heidelberg University, J5, 68159 Mannheim, Germany; 11 Munich Cluster for Systems Neurology (SyNergy), Feodor-Lynen-Str. 17, 81377 Munich, Germany; 12 Institute of Translational Medicine, University of Liverpool, Crown Street, Liverpool L69 3BX, UK; 13 Max-Planck-Institute of Psychiatry, Kraepelinstr. 2-10, 80804 Munich, Germany; 14 Department of Psychiatry and Psychotherapy, University of Bonn, Bonn, Germany

**Keywords:** Bipolar disorder, brain development, cortical complexity, magnetic resonance imaging (MRI), major depressive disorder, polygenic risk, schizophrenia, surface-based morphometry

## Abstract

**Background:**

MRI-derived cortical folding measures are an indicator of largely genetically driven early developmental processes. However, the effects of genetic risk for major mental disorders on early brain development are not well understood.

**Methods:**

We extracted cortical complexity values from structural MRI data of 580 healthy participants using the CAT12 toolbox. Polygenic risk scores (PRS) for schizophrenia, bipolar disorder, major depression, and cross-disorder (incorporating cumulative genetic risk for depression, schizophrenia, bipolar disorder, autism spectrum disorder, and attention-deficit hyperactivity disorder) were computed and used in separate general linear models with cortical complexity as the regressand. In brain regions that showed a significant association between polygenic risk for mental disorders and cortical complexity, volume of interest (VOI)/region of interest (ROI) analyses were conducted to investigate additional changes in their volume and cortical thickness.

**Results:**

The PRS for depression was associated with cortical complexity in the right orbitofrontal cortex (right hemisphere: *p* = 0.006). A subsequent VOI/ROI analysis showed no association between polygenic risk for depression and either grey matter volume or cortical thickness. We found no associations between cortical complexity and polygenic risk for either schizophrenia, bipolar disorder or psychiatric cross-disorder when correcting for multiple testing.

**Conclusions:**

Changes in cortical complexity associated with polygenic risk for depression might facilitate well-established volume changes in orbitofrontal cortices in depression. Despite the absence of psychopathology, changed cortical complexity that parallels polygenic risk for depression might also change reward systems, which are also structurally affected in patients with depressive syndrome.

## Introduction

Major mental disorders are highly heritable. Family studies have estimated the overall phenotype heritability of schizophrenia (SZ), bipolar disorder (BD), and major depressive disorder (MDD) at ~80% (Sullivan, Kendler, & Neale, 2003), ~70% (Edvardsen et al., 2008), and ~30–40% (Sullivan, Neale, & Kendler, 2000), respectively. This suggests a substantial involvement of inherited genetic variants in the etiology of these disorders, which has been confirmed by genome-wide association studies (GWAS). The proportion of variance that has been attributed to common variants (single-nucleotide polymorphisms (SNP) heritability) in current GWAS is estimated around 9% for MDD (Howard et al., 2019; Wray et al., 2018), 17–23% for BD (Stahl et al., 2019), and 25–30% for SZ (Brainstorm Consortium et al., 2018; Pardiñas et al., 2018). It is, however, unclear how these genetic risk factors translate to brain structural and functional changes that can lead up to psychopathology.

Neuroimaging studies have reported brain anatomical changes as potential pathogenic features of major mental disorders (Goodkind et al., 2015; SZ: Haijma et al., 2012; BD: Hanford, Nazarov, Hall, & Sassi, 2016; MDD: Schmaal et al., 2016). However, one major question remaining unanswered is which of the structural abnormalities in patients arise from a shared genetic basis with etiology and which reflect pathogenic factors, progression of mental disorders, or treatment-related mechanisms. While the former might be present before the onset of mental disorders, the latter unfolds during the course of disorder progression. Case–control studies do often not account for such confounding effects of comorbidity, therapeutic effects [psychopharmacological, electroconvulsive therapy, psychotherapy (Enneking, Leehr, Dannlowski, & Redlich, 2020; Mulders et al., 2020)], clinical heterogeneity, or also genetic heterogeneity.

Given the lack of larger studies assessing imaging markers of early brain structural development in relation to genetic risk, our present study strives to investigate cortical complexity (Yotter, Nenadic, Ziegler, Thompson, & Gaser, 2011) in healthy participants (HC). Cortical complexity (CC) is a biomarker that measures the roughness of a surface by quantifying the spatial frequency of cortical shape details and can thus be considered as a measure of gyrification (Di Ieva, Grizzi, Jelinek, Pellionisz, & Losa, 2013). Cortical regions with higher fractional dimension values are shaped more irregularly and consist of more spatial details (Im et al., 2006). Other studies showed that CC is affected by genetic disorders like 22q11 deletion syndrome (Schaer et al., 2008) and William's syndrome (Thompson et al., 2005), but also gender (Awate, Yushkevich, Song, Licht, & Gee, 2010; Luders et al., 2004). However, the heritability of cortical folding varies across different brain areas (Elliott et al., 2018; Grasby et al., 2018; Rogers et al., 2010; Strike et al., 2019).

*In vivo* fetal imaging studies showed that CC increases rapidly during intrauterine brain folding development (Shyu et al., 2011; Wu, Shyu, Chen, & Guo, 2009). After birth, in the first two decades of life, smaller maturational changes in CC were observed (Blanton et al., 2001; Sandu et al., 2014; Sun & Hevner, 2014). By contrast, CC stays relatively stable in adulthood (Cao et al., 2017). Thus, we can use CC as a marker for major maturational processes in the brain that occur mainly throughout fetal and early postnatal life (Armstrong, Schleicher, Omran, Curtis, & Zilles, 1995; Hedderich et al., 2020; Sun & Hevner, 2014).

Cross-sectional case–control studies of cortical folding showed changes in cortical folding in patients suffering from SZ (Nenadic, Yotter, Sauer, & Gaser, 2014; Nesvåg et al., 2014; Palaniyappan & Liddle, 2012; Yotter et al., 2011), BD (Nenadic et al., 2017), and MDD (Depping et al., 2018; Schmitgen et al., 2019). It is unknown, though, how individual molecular genetic risk for major mental disorders shapes early developmental cortical folding.

In the current study, we investigated the impact of molecular genetic risk for SZ, BD, and MDD on cortical folding. By analyzing a healthy control sample, we minimize the effects of mental disorder phenotype expressions, medication, and other factors commonly confounding the studies in patient populations. We hypothesized polygenic risk scores (PRS) to be associated with cortical folding in brain areas that have been implicated in these disorders. In particular, we expect, first, polygenic risk for SZ to be associated with CC changes in parietal and frontal regions (Liu et al., 2017; Nenadic et al., 2014; Palaniyappan, Mallikarjun, Joseph, White, & Liddle, 2011); second, BD polygenic risk to be associated with changes in cortical development in frontal areas as well as in the precuneus (Nenadic et al., 2017); third, polygenic risk for MDD to affect cortical folding in the rostral anterior cingulate cortex, orbitofrontal cortex (OFC), and frontal poles (Han et al., 2017). Additionally, we conducted an analysis with cross-disorder polygenic risk (Cross-Disorder Group of the Psychiatric Genomics Consortium, 2013) to test its potential effects on cortical folding complexity. In order to further characterize potential CC clusters that are significantly associated with PRS for major mental disorders, we conducted follow-up analyses with grey matter volumes and cortical thickness. Therefore, we used region of interest (ROI) and volume of interest (VOI) analyses in those brain areas which were significantly associated with PRS for major mental disorders for the purpose of identifying potential additional changes in other morphological modalities.

## Methods

### Participants and MRI data acquisition

We analyzed data from 580 healthy non-clinical participants from the ongoing FOR2107 study (http://for2107.de/; Kircher et al., 2019). All participants gave written informed consent to a study protocol approved by the Ethics Committees of the Philipps University of Marburg or the University of Münster and received a financial compensation. All subjects underwent a structured clinical interview (SCID-I; Wittchen, Wunderlich, Gruschwitz, & Zaudig, 1997) administered by trained clinical raters that is based on DSM-IV-TR. Besides lifelong absence of mental disorders, additional exclusion criteria were any history of neurological (stroke, tumor, neuro-inflammatory diseases, head-trauma) or other major medical conditions (cancer, chronic autoimmune diseases, infections), a current or previous substance dependence, severe obstetric complications, or an IQ <80 [estimated with the MWT-B (Mehrfachwortschatztest-B), a German equivalent of the Multiple Choice Word Test-B (Lehrl, 1995)]. The participants were recruited through local newspaper advertisements. Subsamples recruited at Münster and Marburg had similar demographics except for a significant difference in mean age [*t*(492.55) = 6.73, *p* = 4.7 × 10^−11^, *d* = 0.55]. For detailed descriptive statistics of the sample, see Table 1.

**Table 1.** Sociodemographic characteristics

	Site											
	Marburg			Münster			Total					
Variable	M	s.d.	*n*	M	s.d.	*n*	M	s.d.	*n*	*t*/χ^2^	df	*p*
Demographics												
Age (y)	34.61	12.55	377	28.14	10.14	203	32.34	12.15	580	6.73	492.55	4.7127 × 10^−11^
Gender (female)	230 (61%)		377	128 (63%)		203	358 (62%)		580	0.16*	1	0.694
Handedness	0.75	0.47	377	0.75	0.44	201	0.75	0.46	578	−0.10	576	0.917

*p* shows significant differences after Bonferroni correction for multiple comparisons (*n* = 3). * indicates that a χ^2^-test has been conducted. Handedness was assessed using the EHI (Oldfield, 1971).

We acquired MRI data in the FOR2107 group at two sites, following a quality assurance protocol (Vogelbacher et al., 2018). In Marburg, MRI data were acquired with a 3T MRI scanner (Tim Trio, Siemens, Erlangen, Germany), using a 12-channel head matrix Rx-coil. In Münster, a 3T MRI scanner (Prisma, Siemens, Erlangen, Germany) and a 20-channel head matrix Rx-coil were used. The MP-RAGE sequence used consisted of 176 sagittal slices with an in-plane field-of-view of 256 mm and a voxel size of 1 × 1 × 1 mm (for further MRI acquisition parameters across sites, see Supplementary material). Before preprocessing, scans were manually checked for the absence of artefacts and anatomical abnormalities by a senior clinician and excluded if necessary.

### MRI data preprocessing

CAT12 (version 1278; Gaser, Dahnke, Kurth, Luders, & Alzheimer's Disease Neuroimaging Initiative, in review) builds on SPM (Penny, Friston, Ashburner, Kiebel, & Nichols, 2011) and includes a pipeline for surface-based morphometry. Using default settings, cortical surfaces were extracted with a spherical harmonics approach (Dahnke, Yotter, & Gaser, 2013), topological correction was applied (Yotter, Dahnke, Thompson, & Gaser, 2011), and surfaces were spherically mapped with a volume-based diffeomorphic DARTEL algorithm (Ashburner, 2007), in order to reparametrize the surfaces into a common coordinate system to allow inter-subject analysis (Yotter, Thompson, & Gaser, 2011). Local surface complexity was estimated utilizing a fractal dimensions approach (Yotter et al., 2011).

All modulated CC datasets were smoothed with a Gaussian kernel of 20 mm full width at half maximum (FWHM). In order to assign significant clusters to anatomical areas, we used the Desikan-Killiany-40 atlas (Desikan et al., 2006).

Additionally, we extracted cortical thickness and grey matter volumes from our MRI data for follow-up analyses (Dahnke et al., 2013). For volume data, structural MRI scans were first spatially registered with a high-dimensional DARTEL template provided by CAT12 to achieve more accurate inter-subject registration. Data were segmented into different tissues (grey matter, white matter, and cerebrospinal fluid) and MRI inhomogeneities were normalized. Segmentations were modulated by scaling with the portion of volume changes due to spatial registration in that way that the total amount of grey matter in the modulated image remains the same as it would be in the original image. For exclusion of artefacts on the grey–white matter border (i.e. incorrect voxel classification), we applied an absolute grey matter threshold of 0.1. Data were then smoothed using a kernel of 8 mm (FWHM). VOIs were selected as anatomical regions that approximately overlap with regions that showed significant associations with CC changes that are significantly associated with PRS for mental disorders. They were defined by using the neuromorphometrics atlas (Neuromorphometrics, Inc., 2019). We analyzed the left and right posterior orbital, anterior orbital, lateral orbital, inferior frontal orbital, and medial orbital gyri.

For cortical thickness estimation, we used a fully-automated method that reconstructs the central surface of the cortex and, thereby, computes the cortical thickness (Dahnke et al., 2013; Yotter, Dahnke, et al., 2011; Yotter, Thompson, et al., 2011). We smoothed the cortical thickness by applying a kernel of 15 mm (FWHM). ROIs were chosen as anatomical regions that approximately overlap with regions that showed significant associations with changes in CC that are significantly associated with PRS for mental disorders. They were defined by the Desikan-Killiany atlas (Desikan et al., 2006). Regions included in our ROI analyses were the left and right lateral orbitofrontal and medial OFC. Homogeneity checks were performed in the CAT12 Toolbox, and all images passed the quality assurance protocol.

### Genotyping, imputation, and PRS calculation

DNA was extracted from peripheral blood samples using standard methods. Genotyping was performed using Illumina Infinium PsychArray-24 BeadChips (Illumina, San Diego, CA, USA). The GenomeStudie software (v.2011.1, Illumina) and the Genotyping Module (v.1.9.4) were used to perform clustering and initial quality control. Subsequent quality control was conducted in PLINK v1.90b5 (Chang et al., 2015) and *R* v3.3.3. Individuals were removed if they met any of the following criteria: genotyping call rate <98%, gender mismatches or other X-chromosome-related issues, genetic duplicates, cryptic relatedness with pi-hat ⩾0.125, genetic outlier with a distance from the mean of >4 standard deviations (s.d.) in the first eight ancestry components, or a deviation of the autosomal or X-chromosomal heterozygosity from the mean >4 s.d. Genotype data were imputed to the 1000 Genomes Phase 3 reference panel using SHAPEIT and IMPUTE2 (Delaneau, Zagury, & Marchini, 2012; Howie, Donnelly, & Marchini, 2009; Howie, Fuchsberger, Stephens, Marchini, & Abecasis, 2012). In order to adjust for population stratification, multi-dimensional scaling (MDS) components were computed based on the pairwise identity-by-state distance matrix, calculated on the genotype data in PLINK. For further details, see the Supplementary methods.

PRS were calculated by summing the minor allele dosage of the LD-independent single nucleotide polymorphisms in the target sample, weighted by different GWAS effect sizes [cross-disorder (overlapping genetic risk for MDD, SZ, BD, autism spectrum disorder, and attention-deficit/hyperactivity disorder): Cross-Disorder Group of the Psychiatric Genomics Consortium, 2013; SZ: Ripke et al., 2014; BD: Stahl et al., 2019; MDD without 23andMe: Wray et al., 2018]. PRS were calculated in *R* v3.33 using imputed genetic data. For each PRS, the effect sizes of variants below a selected *p* value threshold, both obtained from large GWAS (training data), were multiplied by the imputed SNP dosage in the test data and then summed to produce a single PRS per threshold. The PRS thus represent a cumulative, weighted, additive risk. For additional details, see the Supplementary methods. In our analyses, we used PRS with a *p* value threshold of *p* = 5 × 10^−8^ (see Table 2 for intercorrelations of PRS).

**Table 2.** Intercorrelations of polygenic risk scores

Polygenic risk score	MDD	SZ	BD	CROSS	Number of SNPs included in the PRS
MDD	–				6
SZ	0.133* (*p* = 0.001)	–			119
BD	−0.004 (*p* = 0.925)	0.072 (*p* = 0.082)	–		18
CROSS	−0.051 (*p* = 0.223)	0.164* (*p* < 0.001)	0.079 (*p* = 0.057)	–	4

*p* shows significant differences after Bonferroni correction for multiple comparisons (*n* = 6).

### Statistical analysis of associations between PRS for major mental disorders and brain morphology

We conducted separately for each hemisphere and separately for each PRS multiple regressions with CAT12 resulting in eight separate tests. CC was used as regressand, the PRS-variables as regressors, and age, quadratic age, gender, site, and three ancestry components as covariates. Our quality assurance protocol (Vogelbacher et al., 2018) showed non-negligible differences in the quality of MRI images after the replacement of a gradient coil in Marburg that took place after 322 from a total of 377 participants were scanned at this site. We accounted for this in our statistical model by using an additional scanner-covariate. Cognition and brain morphology share genetic influences from liabilities for mental disorders (Toulopoulou et al., 2015). Therefore, potential associations between PRS related to mental disorders and CC could be mediated by cognitive abilities, which are associated with both regional variations in cortical folding (Gautam, Anstey, Wen, Sachdev, & Cherbuin, 2015; Gregory et al., 2016) as well as polygenic risk for major mental disorders in the general population (Clarke et al., 2016; Germine et al., 2016; Mallet, Le Strat, Dubertret, & Gorwood, 2020; Shafee et al., 2018). For this reason, we repeated all multiple regressions with years of education as an additional covariate.

For each multiple regression, we conducted an *F*-contrast and computed for each vertex on the cortex surface the threshold that has been exceeded in order to reach significance. We report results at the initial significance height threshold of *α* = 0.001 and also after applying FWE-correction at the significance height threshold *α* = 0.05 based on Gaussian random field theory to adjust for multiple testing of each vertex (Nichols & Hayasaka, 2003). To correct for multiple testing in the FWE-analyses, we divided *α* by the eight conducted tests, which results in *α* = 0.00625. For every FWE-significant association, we calculated the coefficient of determination *R*^2^. Therefore, we extracted predicted *β*-values from uncorrected clusters that withstood FWE-correction that are based on the contrast of the corresponding multiple regression, including its covariates and residuals using the CAT12 function *cat_surf_results*.

Association analyses for grey matter volumes of interest and cortical thickness regions of interest were identical, except total intracranial volume was used as an additional covariate in the analyses of grey matter volumes. We set the initial significance level at *α* = 0.05. To correct for multiple testing resulting from the 14 ROIs/VOIs, we adjusted the threshold to *α* = 0.0036.

## Results

### Associations between polygenic risk for MDD and CC

**Fig. 1.**
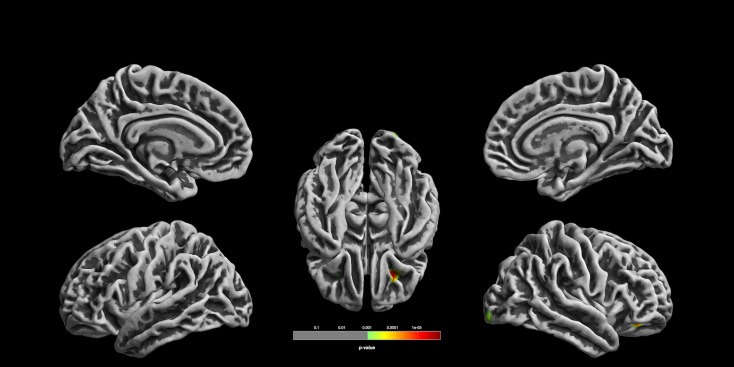
Associations between the polygenic risk for MDD and CC. Orbitofrontal cortical folding complexity is significantly associated with polygenic risk for major depression (for the purpose of display, images are shown at *p* < 0.001, uncorrected threshold). The cluster in 24/33/-12 withstood correction for multiple comparisons (*p* = 0.006, FWE cluster-level correction).

**Fig. 2.**
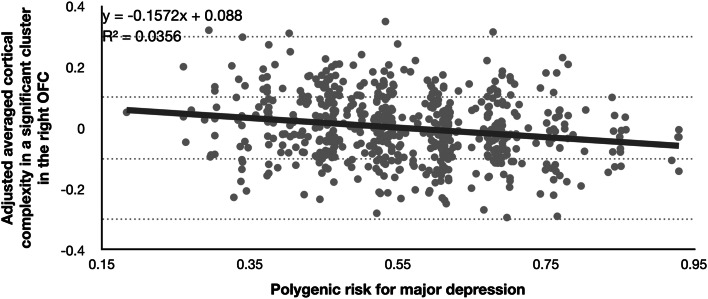
Scatter plot showing the association between the polygenic risk score for MDD and adjusted averaged cortical complexity in a significant cluster in the right orbitofrontal cortex. *Note*. Adjusted cortical complexity values were cluster-wise extracted for every participant using the CAT12 function *cat_surf_results*. Cluster values were calculated as *β*-values based on the used contrast of the corresponding multiple regression, including its covariates and residuals. A non-parametric correlation yielded also a significant association: Spearman's *ρ* = −0.189 (*p* < 0.0001).

We found a significant association with PRS for MDD in the right OFC that withstood correction for multiple testing (*k* = 453, *F* = 21.69, *p* = 0.0000039, uncorrected, *R*^2^ = 0.036; *p* = 0.006, FWE-corrected; see Table 3, Figs 1 and 2). In exploratory follow-up analyses without correction for multiple testing, we observed nominally significant associations contralateral in the left OFC (*k* = 20, *F* = 11.15, *p* = 0.000897, uncorrected, *p* = 0.606, FWE-corrected) and also in the right lateral occipital cortex (*k* = 133, *F* = 12.7, *p* = 0.0003968, uncorrected, *p* = 0.364, FWE-corrected). In follow-up VOI/ROI-analyses with grey matter volumes and cortical thickness, there were no significant associations with the PRS for MDD (all *p* > 0.0036; see Supplementary material).

**Table 3.** Overview of associations between polygenic risk scores and cortical complexity

	Coordinates			Anatomical region according to DK-40	*k*	*F*	*p* (*α* = 0.001)	*p* (FWE) (*α* = 0.00625)
**Polygenic risk for major depressive disorder**								
Left hemisphere	−28	33	−12	Orbitofrontal	20	11.15	0.000897	0.606
Right hemisphere	**24**	**33**	**−12**	**Orbitofrontal**	**453**	**21.69**	**0.0000039**	**0.006**
	28	−98	−9	Lateral occipital	133	12.7	0.0003968	0.364
**Polygenic risk for cross-disorder**								
Left hemisphere	−36	26	43	Caudal middle frontal	67	11.71	0.0006657	0.515
Right hemisphere	49	−78	4	Lateral occipital	234	16.96	0.0000437	0.063
**Polygenic risk for schizophrenia**								
Left hemisphere	–	–	–	No suprathreshold clusters	–	–	–	–
Right hemisphere	12	30	−26	Lateral orbitofrontal	121	12.36	0.0004744	0.411
	28	−60	4	Lingual (93%), precuneus (7%)	69	11.81	0.0006327	0.491
**Polygenic risk for bipolar disorder**								
Left hemisphere	–	–	–	No suprathreshold clusters	–	–	–	–
Right hemisphere	–	–	–	No suprathreshold clusters	–	–	–	–

Note *p* and *p* (FWE) are shown at cluster-level and *k* refers to the cluster size at uncorrected thresholds. Significance thresholds were set at *α* = 0.001 and *α* = 0.00625 when correcting for multiple testing. Multiple regressions were performed using the following covariates: age, quadratic age, gender, site, MRI scanner, and three MDS-components. Bold indicates statistically significant results after applying FWE-correction. Cluster labeling was executed with the Desikan-Killiany-40 atlas (Desikan et al., 2006).

### Associations between polygenic risk for SZ and CC

The general linear model including the PRS for SZ showed no significant associations when correcting for multiple testing. In the exploratory analysis, we found a nominally significant association between the PRS for SZ and CC in a cluster also located in the right OFC (*k* = 121, *F* = 12.3, *p* = 0.0004744, uncorrected, *p* = 0.411, FWE-corrected). Additionally, we found a nominally significant cluster ranging over the right lingual gyrus (93%) and the right precuneus (7%; *k* = 69, *F* = 11.81, *p* = 0.0006327, uncorrected, *p* = 0.491, FWE-corrected).

### Associations between polygenic risk for BD and CC

There was no significant association between the polygenic risk for BD and CC in our sample.

### Associations between cross-disorder polygenic risk and CC

We found no significant association between the cross-disorder PRS and CC when applying FWE-correction. Exploratory analysis with uncorrected thresholds showed a nominally significant association in a cluster in the right lateral occipital cortex (*k* = 234, *F* = 16.96, *p* = 0.0000437, uncorrected, *p* = 0.063, FWE-corrected). Our analyses also revealed a nominally significant association in the left caudal middle frontal cortex (*k* = 67, *F* = 11.71, *p* = 0.0006657, uncorrected, *p* = 0.515, FWE-corrected).

Statistical results of the conducted multiple regressions changed only marginally when we included years of education as an additional covariate (see Supplementary material).

## Discussion

In the current study, we characterized the impact of SNP-related genetic risk for SZ, MDD, BD, and cross-disorder on cortical folding complexity in a large sample of healthy subjects. CC is a marker reflecting prenatal and early postnatal brain development (Nenadic et al., 2014, 2017). Our main finding is the association between MDD polygenic risk and CC in the OFC. We show that early cortical folding is associated with polygenic risk for major mental disorders, which potentially predisposes for later expressions of psychopathology.

### Association between CC and polygenic risk for depression

We found an association between MDD polygenic risk and CC in the OFC, a key anatomical region in the pathophysiology of this disorder. Previous analyses in MDD patients have observed decreased (Depping et al., 2018; Zhang et al., 2009), as well as increased (Han et al., 2017) local gyrification in the orbitofrontal gyrus to be linked to the expression of the phenotype of this mental disorder, while yet others found no changes in gyrification in the OFC in MDD (Peng et al., 2015; Schmitgen et al., 2019). Therefore, the observed association between altered CC in the OFC in HC and MDD polygenic risk might constitute only one specific intermediary phenotype that is associated with vulnerability for MDD and might not be observed across all patients with depression. This emphasizes the multiple exposure pathways of genetic risk for MDD and also the current assumption that the depressive syndrome reflects on a nosological level a heterogeneity of different mental disorders (Drysdale et al., 2017; Insel & Cuthbert, 2015). Our work might therefore help to further disaggregate the complex phenotype depression for refinements of taxonomies of mental disorders.

Several recent studies with large sample sizes and meta-analyses that investigated grey matter volumes and cortical thickness consistently reported reductions in the OFC in MDD patients (Arnone, McIntosh, Ebmeier, Munafò, & Anderson, 2012; Koolschijn, van Haren, Lensvelt-Mulders, Hulshoff Pol, & Kahn, 2009; Schmaal et al., 2016; Suh et al., 2019). These well-documented structural changes could be facilitated by genetically induced disrupts in early cortical development that are reflected in CC during MDD expression.

It is not yet fully understood to which extent these cortical volume and thickness reductions in the OFC are a result of pathogenic factors and to which extent they reflect genetic effects that can also be found in HC. In order to empirically address this question, we executed VOI/ROI-analyses in the OFC. Since we use a healthy control sample, we are able to preclude effects from phenotype expression of this mental disorder and treatment. Our results showed no significant associations neither between the PRS for MDD and grey matter volume nor between the PRS for MDD and cortical thickness. Thus, the structural changes in grey matter volume and cortical thickness in the OFC found in MDD patients do not precede pathogenic processes as a result of high additive genetic risk for MDD. This hypothesis is further empirically supported by studies investigating grey matter volumes and cortical thickness of the OFC in drug-naïve (but not always treatment-naïve) MDD patients. A meta-analysis and other VBM studies on medication-naïve first-episode MDD patients found no volumetric changes in the OFC relative to HC (Kong et al., 2014; Shen et al., 2016; Zhao et al., 2014). Additionally, a study on cortical thickness showed evidence for no changes in cortical thickness between HC and drug-naïve MDD patients (Peng et al., 2015), but some others did not (Qiu et al., 2014; Shen et al., 2019).

Volume changes in the OFC in MDD patients are modifiable through a wide range of therapeutic interventions (Gbyl et al., 2019; Kong et al., 2014; Mackin et al., 2013; Phillips, Batten, Tremblay, Aldosary, & Blier, 2015; van Eijndhoven et al., 2013). Potentially, these structural changes interact with CC changes associated with high genetic burden, and therefore CC alterations may not only facilitate disorder outbreak but also influence therapeutic responses.

Chronic MDD patients and patients with relapse also show volume changes in the OFC (Frodl et al., 2008; Schmaal et al., 2016; Zaremba et al., 2018). In the context of the results presented in this study, one could suggest that chronicity and relapse are influenced by a high genetic burden. Potentially, genetically induced CC changes in the OFC prevent volume modifications associated with treatment which complicates recovery. Simultaneously, chances of relapse are increased by CC changes that facilitate grey matter volume reductions and cortical thinning. Further studies could investigate whether chronic MDD patients and patients with relapse also suffer from higher additive genetic risk.

We conclude that genetically determined liability for MDD potentially impacts on the OFC development which is primarily defined prenatally and during early life. This might increase vulnerability for a broad range of morphological changes associated with a higher MDD risk, but also therapeutic response, chronicity, and relapse. Overall, this emphasizes the importance of the OFC as a biomarker for MDD.

Changes in the folding of the OFC in HC might additionally lead to functional changes in this brain area, for which there is considerable evidence in MDD patients. The non-reward attractor theory proposes that non-reward systems which are located in the OFC are over-responsive in MDD (Groenewold, Opmeer, de Jonge, Aleman, & Costafreda, 2013; Rolls, 2016, 2019). Functional connectivity is increased in the lateral OFC in MDD and the reward-involved medial OFC shows decreased functional connectivity. Thus, less CC in the OFC, which could be a consequence of polygenic risk for MDD, might moderate interindividual differences in reward processing. This genetically induced cognitive change that is moderated by CC changes could be a behavioral manifestation of increased vulnerability to MDD and ease potential later pathogenic developments that change functional connectivity in MDD.

According to the tension-based hypothesis, cortical convolution during brain development is influenced by axonal tension leading to either elongation or retraction and thereby forming gyri and sulci (Hilgetag & Barbas, 2006; Kroenke & Bayly, 2018; Xu et al., 2010; Zilles, Palomero-Gallagher, & Amunts, 2013). Thus, the folding of the cortex can to a certain extend be explained by the underlying white matter connectivity and any changes in it can be interpreted as a result of disruptions in the connectivity of underlying axons. In this context, we could conclude that the genetically induced variations in OFC folding shown by this study potentially arise from genetically induced changes in inter-regional connectivity. This notion is consistent with empirical support from a diffusion tensor imaging study finding changes in white matter tracts such as the superior longitudinal fasciculi, inferior fronto-occipital fasciculi, corpus callosum, and thalamocortical radiations in MDD patients as well as HC at high risk for MDD that also connects with the OFC (Whalley et al., 2013).

### Association between CC and cross-disorder polygenic risk

Our analyses showed no associations between the used PRS for cross-disorders and CC. However, there was a trend for such a relationship in the right lateral occipital cortex (*p* = 0.063, FWE-corrected), which we discuss cautiously due to its questionable statistical validity. Although this region has mainly been associated with visual stimulus processing, recent research has shown that resilience is associated with changes in cortical thickness in the lateral occipital cortex (Kahl, Wagner, de la Cruz, Köhler, & Schultz, 2020), which might be a result of genetically-induced differential cortical folding. The association could also be explained by occipital bending, a pattern of curvature in the brain whereby one occipital lobe wraps around the other which has been observed in MDD (Fullard et al., 2019; Maller et al., 2014), SZ (Deutsch, Hobbs, Price, & Gordon-Vaughn, 2000; Maller et al., 2016), and BD (Maller et al., 2015). It has been hypothesized that occipital surface variation might be a neurobiological variation that signals an increased vulnerability to major mental disorders in general (Koch & Schultz, 2014).

### No association between CC and polygenic risk for SZ

There were no associations between the applied PRS for SZ and CC at the chosen conservative statistical threshold level. Other studies, however, showed dynamic expressions of genes associated with SZ during fetal development and early infancy in the prefrontal cortex (Clifton et al., 2019). Additionally, associations between polygenic risk for SZ and gyrification in the inferior parietal lobules (Liu et al., 2017) are mentioned in the literature, but no associations with surface area (Neilson et al., 2019), although both biomarkers are affected by cortical folding.

It is worthwhile mentioning that we were able to demonstrate an association between CC and the PRS for SZ in the right OFC, when not controlling for multiple testing (*p* < 0.001, uncorr.). Additionally, this significant CC-cluster is regionally partly overlapping with the one we found when investigating associations between CC and the PRS for MDD when applying FWE-correction. Since both PRS are intercorrelated (Table 2) and both include partly the same SNPs, we can assume that CC in the OFC might not be specific for either polygenic risk for MDD nor SZ. This means that not only genetic etiology is to a certain extent overlapping in both MDD and SZ, but that there might potentially also be an overlap between the different neurobiological risk phenotypes for these mental disorders.

The lack of significant associations in our sample could also be due to a lack of statistical power if effects were less focal. Remarkably, the CC changes associated with polygenic risk for SZ at uncorrected thresholds in this study partially overlap with findings from clinical studies on SZ patients (Nenadic et al., 2014; Nesvåg et al., 2014; Yotter et al., 2011).

Compared to the used MDD PRS, the PRS for SZ includes a larger number of variants and thus implicated genes. Therefore, it likely reflects potential consequences on a broader range of biological pathways. As some of them are potentially not affecting cortical folding, future studies might explore which genes influence CC. This would enable studies that use PRS that only include genetic risk variants for mental disorders from which we already know that they affect the folding of the cortex (Spalthoff et al., 2019).

### Association between CC and polygenic risk for BD

There were no significant associations between the polygenic risk for BD and CC applying a stringent statistical threshold. This would suggest that cortical folding abnormalities observed in BD (Nenadic et al., 2017) might arise mainly from environmental or other pathological effects.

Additionally, genetically transmitted abnormalities in CC in HC could be limited to subgroups since different clinical phenotypes (e.g. age at onset, with *v.* without psychosis) are associated with different cortical folding patterns (Sarrazin et al., 2018). These different neurodevelopmental subtypes are often interpreted as reflections of underlying genetic heterogeneity in BD (Kalman et al., 2019; Lin et al., 2006).

### Different directions of effects

The effects on CC arising from polygenic risk for three major mental disorders and cross-disorder point to different directions of effects, i.e. both subtle hypo- and hypergyrification. This aspect is consistent with findings in patients, in which both increases and decreases of cortical folding parameters are found in the same samples (Nenadic et al., 2014, 2017; Palaniyappan & Liddle, 2012; Yotter et al., 2011). Therefore, significant deviations from the mean in either direction might serve as an indicator for subsequent risk for psychopathology. However, we also need to consider the possibility that parts of the variation observed in our analyses are related to resilience, as none of our adult healthy controls had experienced a mental disorder.

### Limitations

We only analyzed a cumulative SNP-based genetic risk burden, which does not include gene-interaction effects that influence the risk for a particular disorder phenotype, and also does not take into account rare genetic variants such as copy number variants.

In this study, we used only SNPs that showed genome-wide significance (*p* = 5 × 10^−8^). Thereby, we wanted to focus only on the SNPs showing the strongest statistical support for an association with disorder risk. Consequently, our PRS represent only a limited amount of the polygenic risk background for the disorders.

Between-individual variability in brain folding that depends on polygenic risk is determined by gene–environment interactions, and therefore, also reflects differences in sensitivity to environmental and genetic perturbations. The analysis of HC may mask considerable environmental contributions to brain development and may result in less heterogeneity not depicting the whole spectrum.

Environmental factors such as paternal education and maternal ethnicity also act *in utero* on cortex development (Girault et al., 2018). Additionally, it has been demonstrated that potential environmental risk factors during pregnancy such as smoking, age at delivery, pre-pregnancy body mass index, and use of acetaminophen during the second half of pregnancy are associated with maternal risk alleles, primarily maternal polygenic risk for ADHD (Leppert et al., 2019). It should thus be taken into consideration that possibly maternal genetic factors are confounding our results, which makes inferring causal relationships impossible.

## Conclusion

In conclusion, this study provides novel insights into how cumulative genetic influences shape cortical structure during brain development. We argue that CC changes in the OFC in HC that are significantly associated with polygenic risk for MDD precede disorder expression. However, this additive genetic risk is not associated with reduced grey matter volume and cortical thinning in HC, which is a robust finding in MDD patients. CC aberrations in HC associated with disorder-related polygenic risk could therefore facilitate well-researched morphological changes in the OFC associated with MDD during disorder expression.

Further, we propose that the demonstrated CC alterations in HC that tend to parallel polygenic risk for MDD might change non-reward systems in HC which are structurally changed in MDD patients. Future research focusing on relationships between MDD and CC, using a spherical harmonics approach, could aid the detection of pathogenic effects on CC and thereby further characterize the neurobiological correlates of the courses of this mental disorder.

## Supplementary material

For supplementary material accompanying this paper visit https://doi.org/10.1017/S0033291721001082. click here to view supplementary material
