# Supplementary material for: Effects of polygenic risk for major mental disorders and cross-disorder on cortical complexity
Source: Psychol Med. 2021 Apr 8;52(16):4127–38. doi: 10.1017/S0033291721001082 (PMC9811276; doi:10.1017/S0033291721001082)
Supplement: Supplementary file 1 [file S0033291721001082sup001.docx]

Supplement: Effects of polygenic risk for major mental disorders and cross-disorder on cortical complexity

Simon Schmitt, M.Sc.^1,2,3^, Tina Meller, Ph.D.^1,2^, Frederike Stein M.A.^1,2^, Katharina Brosch, M.Sc.^1,2,3^, Kai Ringwald, M.Sc.^1,2^, Julia-Katharina Pfarr, M.Sc.^1,2^, Clemens Bordin^1^, Nina Peusch^1^, Olaf Steinsträter, Ph.D.^1^, Dominik Grotegerd, Ph.D.^4^, Katharina Dohm, Ph.D.^4^, Susanne Meinert, M.Sc.^4^, Katharina Förster, Ph.D.^4^, Ronny Redlich, Ph.D.^4,13^, Nils Opel, M.D.^4^, Tim Hahn, Ph.D.^4^, Andreas Jansen, Ph.D.^1,2,5^, Andreas J. Forstner, M.D.^6,7,14^, Fabian Streit, Ph.D.^8^, Stephanie H. Witt, Ph.D.^8^, Marcella Rietschel, M.D.^8^, Bertram Müller-Myhsok, M.D.^9,10,11^, Markus M. Nöthen, M.D.^7^, Udo Dannlowski, M.D., Ph.D.^4^, Axel Krug, Ph.D.^1,2,12^, Tilo Kircher, M.D.^1,2,3^, Igor Nenadić, M.D.^1,2,3^

^1^ Department of Psychiatry and Psychotherapy, Philipps-Universität Marburg, Rudolf-Bultmann-Str. 8, 35039 Marburg, Germany

^2^ Center for Mind, Brain and Behavior (CMBB), Philipps-Universität Marburg and Justus Liebig Universität Giessen, Hans-Meerwein-Str. 6, 35032 Marburg, Germany

^3^ Marburg University Hospital – UKGM, Rudolf-Bultmann-Str. 8, 35039 Marburg, Germany

^4^ Department of Psychiatry, Westfälische Wilhelms-Universität Münster, Albert-Schweitzer-Campus 1, Building A9, 48149 Münster, Germany

^5^ Core-Facility BrainImaging, Faculty of Medicine, Philipps-Universität Marburg, Rudolf-Bultmann-Str. 8, 35039

^6^ Centre for Human Genetics, Philipps-Universität Marburg, Baldingerstr., 35033 Marburg, Germany

^7^ Institute of Human Genetics, University of Bonn, School of Medicine & University Hospital Bonn, Venusberg-Campus 1, 53127 Bonn, Germany

^8^ Department of Genetic Epidemiology in Psychiatry, Central Institute of Mental Health, Medical Faculty Mannheim, Heidelberg University, J5, 68159 Mannheim, Germany

^9^ Munich Cluster for Systems Neurology (SyNergy), Feodor-Lynen-Str. 17

81377 Munich, Germany

^10^ Institute of Translational Medicine, University of Liverpool, Crown Street, Liverpool L69 3BX, UK

^11^ Max-Planck-Institute of Psychiatry, Kraepelinstr. 2-10, 80804 Munich, Germany

^12^ Department of Psychiatry and Psychotherapy, University of Bonn, Bonn, Germany

^13^ Department of Psychology, University of Halle, Halle, Germany

^14^ Institute of Neuroscience and Medicine (INM-1), Research Center Jülich, Wilhelm-Johnen-Straße, 52428 Jülich, Germany

Corresponding author:

Igor Nenadić

Department of Psychiatry and Psychotherapy, Philipps-Universität Marburg, Rudolf-Bultmann-Str. 8, 35039 Marburg, Germany

Phone: +49 6421 58 65002

Fax: +49 (0)6421 58 68939

Email: nenadic@staff.uni-marburg.de

**Supplementary material**

1. MRI data acquisition across sites

| Table S1  *MRI acquisition parameters across sites* | | | | |
| --- | --- | --- | --- | --- |
| Site | TR | TE | Flip angle | Acquisition duration |
| Münster | 2130 ms | 2.28 ms | 8° | 4:58 min |
| Marburg | 1900 ms | 2.26 ms | 9° | 4:26 min |

2. Genotype quality control (QC)

QC of genotype data was conducted in PLINK v1.90b5 and *R* v3.3.3.

*Sequence of QC steps:*

Before QC: 1,786 individuals and 603,132 variants

- 1. Removal of SNPs with call rates <98% or a MAF <1%
  2. Removal of individuals with genotyping rates <98% (36 removed)
  3. Removal of gender mismatches and other X chromosome-related issues (6 removed)
  4. Removal of genetic duplicates (4 removed)
  5. Removal of cryptic relatives with pi-hat≥12.5 (61 removed)
  6. Removal of genetic outliers with a distance from the mean of >4 SD in the first eight MDS ancestry components (62 removed)
  7. Removal of individuals with a deviation of the autosomal or X-chromosomal heterozygosity from the mean >4 SD (5 removed)
  8. Removal of non-autosomal variants
  9. Removal of SNPs with call rates <98% or a MAF <1% or Hardy-Weinberg Equilibrium (HWE) test p-values <1×10^-6^
  10. Removal of A/T and G/C SNPs
  11. Update of variant IDs and positions to the IDs and positions in the 1000 Genomes Phase 3 reference panel
  12. Alignment of alleles to the reference panel
  13. Removal of duplicated variants and variants not present in the reference panel

After QC: 1,673 individuals and 287,381 variants

*Imputation of genotype data*

Genotypes were aligned to the 1000 Genomes Phase 3 reference panel using SHAPEIT v2 (r837) and PLINK v1.90b5. Pre-phasing (haplotype estimation) was conducted for each chromosome separately using SHAPEIT. Imputation was performed using IMPUTE2 v2.3.2 in 5 Mbp chunks with 500 kbp buffers, filtering out variants that are monomorphic in the EUR samples. Chunks with <51 genotyped variants or concordance rates <92 % were fused with neighboring chunks and re-imputed. Imputed variants with a MAF <1% or an INFO metric <0.8 were removed. Imputed variants in the combined sample after QC: 8,578,636.

*Calculation of ancestry components*

For the calculation of ancestry components (population stratification), pre-imputation genotype data was used, after the QC steps explained above had been applied. Additional variant filtering steps were: removal of variants with a MAF <0.05 or HWE *p*-value <10^-3^; removal of variants mapping to the extended MHC region (chromosome 6, 25-35 Mbp) or to a typical inversion site on chromosome 8 (7-13 Mbp); LD pruning (PLINK command --indep-pairwise 200 100 0.2).

Next, the pairwise identity-by-state (IBS) matrix of all individuals was calculated using the command --genome on the filtered genotype data. Multidimensional scaling (MDS) analysis was performed on the IBS matrix using the eigendecomposition-based algorithm in PLINK v1.90b5.

*Generation and analysis of PRS*

The GWAS test statistics and imputed variants in our data were merged based on chromosome, position, and alleles of each variant. Summary statistics were then clumped in PLINK v1.90b5.2, based on best-guess genotype data (hard-call threshold 0.3) using the following parameters:

--clump-kb 500 --clump-r2 0.1 --clump-p1 1 --clump-p2 1

PRS were then calculated in R v.3.3 based on imputed (dosage) data. Test statistics and alleles in the GWAS training data were flipped so that effect sizes were always positive. Thus, the weighted PRS represent cumulative, additive risk. PRS were scaled to represent the relative risk load (minimum possible cumulative risk load = 0, maximum = 1). For each disorder PRS were calculated using the *p*-value threshold <5×10-8.

**3. Scree plot of the relative genetic variance explained by MDS ancestry components**

**4. Table S2: Associations between polygenic risk scores for MDD and the brain morphological markers grey matter volume and cortical thickness in the orbitofrontal cortex.**

| Table S2 | | |
| --- | --- | --- |
| VOI/ROI | *F* | *p* |
| Grey matter volumes |  |  |
| Left posterior orbital gyrus | 0.000842449 | 0.976855 |
| Left anterior orbital gyrus | 0.371311 | 0.542534 |
| Left lateral orbital gyrus | 0.614774 | 0.433322 |
| Left inferior frontal orbital gyrus | 2.40216 | 0.121724 |
| Left medial orbital gyrus | 3.55521 | 0.059868 |
| Right posterior orbital gyrus | 0.0814233 | 0.775482 |
| Right anterior orbital gyrus | 3.02285 | 0.08264 |
| Right lateral orbital gyrus | 0.0734746 | 0.786442 |
| Right inferior frontal orbital gyrus | 0.61689 | 0.432532 |
| Right medial orbital gyrus | 4.93025 | 0.0267837 |
|  |  |  |
| Cortical thickness |  |  |
| Left lateral orbitofrontal | 0.0752088 | 0.783998 |
| Left medial orbitofrontal | 0.0220629 | 0.881972 |
| Right lateral orbitofrontal | 0.200534 | 0.65446 |
| Right medial orbitofrontal | 0.0935414 | 0.759834 |

*Note.* We set the initial significance level at ⍺ = 0.05. To correct for multiple testing (*n* = 14), we adjusted the threshold to ⍺ = 0.0036. Multiple Regressions were performed using the following covariates: age, quadratic age, gender, site, MRI scanner and three MDS-components. VOI/ROIs were defined according to the definitions in the neuromorphometrics atlas for grey matter volumes (Neuromorphometrics, 2019) and with the Desikan-Killiany atlas for cortical thickness data, respectively (Desikan et al., 2006).

***5. Table S3: Overview of associations between polygenic risk scores and cortical complexity when controlling for years of education.***

| Table S3  *Overview of associations between polygenic risk scores and cortical complexity* | | | | | | | | |
| --- | --- | --- | --- | --- | --- | --- | --- | --- |
|  | coordinates | | | anatomical region  according  to DK-40 | *k* | *F* | *p*  (⍺ = 0.001) | *p* (FWE) *(*⍺ = 0.00625*)* |
| **Polygenic Risk for Major Depressive Disorder** | | | | | | | | |
| left hemisphere | -25 | 29 | -16 | orbitofrontal | 59 | 11.57 | 0.000718 | 0.538 |
| right hemisphere | **22** | **29** | **-13** | **orbitofrontal** | **459** | **21.78** | **0.0000028** | **0.006** |
|  | 31 | -93 | -10 | lateral occipital | 127 | 12.63 | 0.0004104 | 0.373 |
| **Polygenic Risk for Cross-Disorder** | | | | |  |  |  |  |
| left hemisphere | -36 | 30 | 42 | caudal middle frontal | 83 | 11.92 | 0.0006967 | 0.483 |
| right hemisphere | 51 | -70 | 5 | lateral occipital | 245 | 17.19 | 0.0000389 | 0.057 |
| **Polygenic Risk for Schizophrenia** | | | | |  |  |  |  |
| left hemisphere | - | - | - | no suprathreshold clusters | - | - | - | - |
| right hemisphere | 11 | 26 | -22 | lateral orbitofrontal | 113 | 12.22 | 0.0005083 | 0.429 |
|  | 25 | -55 | 3 | lingual (93%), precuneus (7%) | 90 | 12.06 | 0.0005536 | 0.453 |
| **Polygenic Risk for Bipolar Disorder** | | | | |  |  |  |  |
| left hemisphere | - | - | - | no suprathreshold clusters | - | - | - | - |
| right hemisphere | - | - | - | no suprathreshold clusters | - | - | - | - |

Note p and p (FWE) are shown at cluster-level and k refers to the cluster size at uncorrected thresholds. Significance thresholds were set at ⍺ = 0.001 and ⍺ = 0.00625 when correcting for multiple testing. Bold indicates statistically significant results after applying FWE-correction. Multiple Regressions were performed using the following covariates: age, quadratic age, gender, years of education, site, MRI scanner and three MDS-components. Cluster labelling was executed with the Desikan-Killiany-40 Atlas (Desikan et al., 2006).

**6. Table S4: Associations between polygenic risk scores for MDD and the brain morphological markers grey matter volume and cortical thickness in the orbitofrontal cortex when controlling for years of education.**

| Table S4 | | |
| --- | --- | --- |
| VOI/ROI | *F* | *p* |
| **Grey matter volumes** |  |  |
| Left posterior orbital gyrus | 0.192025 | 0.661404 |
| Left anterior orbital gyrus | 0.38749 | 0.533874 |
| Left lateral orbital gyrus | 0.629514 | 0.427868 |
| Left inferior frontal orbital gyrus | 2.839501 | 0.092527 |
| Left medial orbital gyrus | 4.502478 | 0.034282 |
| Right posterior orbital gyrus | 0.247054 | 0.619351 |
| Right anterior orbital gyrus | 3.221162 | 0.073228 |
| Right lateral orbital gyrus | 0.099528 | 0.752514 |
| Right inferior frontal orbital gyrus | 0.648678 | 0.420926 |
| Right medial orbital gyrus | 5.467917 | 0.019718 |
|  |  |  |
| **Cortical thickness** |  |  |
| Left lateral orbitofrontal | 0.933363 | 0.334401 |
| Left medial orbitofrontal | 0.105021 | 0.746005 |
| Right lateral orbitofrontal | 0.005887 | 0.938869 |
| Right medial orbitofrontal | 0.036283 | 0.849001 |

*Note.* We set the initial significance level at ⍺ = 0.05. To correct for multiple testing (*n* = 14), we adjusted the threshold to ⍺ = 0.0036. Multiple Regressions were performed using the following covariates: age, quadratic age, gender, years of education, site, MRI scanner and three MDS-components. VOI/ROIs were defined according to the definitions in the neuromorphometrics atlas for grey matter volumes (Neuromorphometrics, 2019) and with the Desikan-Killiany atlas for cortical thickness data, respectively (Desikan et al., 2006).

**References**

Desikan, R. S., Ségonne, F., Fischl, B., Quinn, B. T., Dickerson, B. C., Blacker, D., . . . Hyman, B. T. J. N. (2006). An automated labeling system for subdividing the human cerebral cortex on MRI scans into gyral based regions of interest. *NeuroImage, 31*(3), 968-980.

Neuromorphometrics, I. (2019). Neuromorphometrics, Inc. Building a Model of the Living Human Brain. Retrieved from <http://www.neuromorphometrics.com>
